# In-cell Solid-State NMR Studies of Antimicrobial Peptides

**DOI:** 10.3389/fmedt.2020.610203

**Published:** 2020-12-17

**Authors:** Frances Separovic, David W. Keizer, Marc-Antoine Sani

**Affiliations:** ^1^School of Chemistry, University of Melbourne, Melbourne, VIC, Australia; ^2^Bio21 Molecular Science and Biotechnology Institute, University of Melbourne, Melbourne, VIC, Australia

**Keywords:** antimicrobial peptides, bacteria, solid-state NMR, in-cell NMR, mode of action

## Abstract

Antimicrobial peptides (AMPs) have attracted attention as alternatives to classic antibiotics due to their expected limited pressure on bacterial resistance mechanisms. Yet, their modes of action, in particular *in vivo*, remain to be elucidated. *In situ* atomistic-scale details of complex biomolecular assemblies is a challenging requirement for deciphering the complex modes of action of AMPs. The large diversity of molecules that modulate complex interactions limits the resolution achievable using imaging methodology. Herein, the latest advances in in-cell solid-state NMR (ssNMR) are discussed, which demonstrate the power of this non-invasive technique to provide atomic details of molecular structure and dynamics. Practical requirements for investigations of intact bacteria are discussed. An overview of recent *in situ* NMR investigations of the architecture and metabolism of bacteria and the effect of AMPs on various bacterial structures is presented. In-cell ssNMR revealed that the studied AMPs have a disruptive action on the molecular packing of bacterial membranes and DNA. Despite the limited number of studies, in-cell ssNMR is emerging as a powerful technique to monitor *in situ* the interplay between bacteria and AMPs.

## Introduction

Antimicrobial peptides (AMPs) are found in all living organisms, serving as an arsenal against pathogens and as modulators of the host immune system. These peptides serve as sentinels but are also produced in response to infectious and inflammatory stimuli (1). Thousands of AMPs have been discovered [APD (http://aps.unmc.edu/AP/)], mainly isolated from evolved immune systems that need to cohabit with a large array of pathogens (i.e., other cells) some have been designed *in silico* and many remain to be discovered. Indeed, new AMPs are constantly being investigated as the search for potent antimicrobial therapeutics is a high priority for providing alternatives as antibiotic resistance increases. Resistance also evolves naturally as cohabitation between microorganisms leads to competition for and production of antibiotics. A pressing problem is acquired-resistance in hospital environments, responsible for millions of premature deaths and a trillion dollar cost to the global economy (http://amr-review.org/). The acquired-resistance phenomenon can be seen as an accelerated selection process where pathogens have either mutated or incorporated genetic material to survive an increased antibiotic pressure. The difficult task of managing antibiotic-resistance in clinical practice is complex. For instance, some infected patients may tolerate higher antibiotic levels to overcome the resistance, while others could develop health threatening side-effects. Likewise, the level of drug that is actually taken up at the site of infection, particularly for deep-seated pathogens, can differ between *in vitro* and *in vivo* environments (2). Overall, these scenarios stress that it is essential to study antimicrobial agents in their inherent environment so as to understand their mechanism of action.

AMPs have been considered as a potent alternative to antibiotics because they act fast, exploit a specific affinity for bacterial lipid membranes and often have multiple intracellular targets, which together are likely to reduce the development of AMP resistance. However, very few AMPs have reached the clinic, partly due to the pharmacokinetic discrepancies between their *in vitro* and *in vivo* behaviors (3), limiting systemic administration and instead favoring topical applications. AMPs range from *ca*. 10 to over 40 amino acids long with mainly cationic and amphipathic properties, which unifies them in targeting negatively charged lipid membranes, and often lead to bilayer disruption (4). The molecular mechanism of action is thought to be correlated with the structure of AMPs when in contact with the pathogens; thus structure elucidation techniques are key to the determination of critical structural features that modulate the potency of AMPs *in situ*. The ability to extract atomic details of molecular interactions within functioning cells is the utmost challenge in biology. There are many challenges to face when in-cell studies are performed, from the lifetime of the cells to the resolution of the signal.

## Structure Elucidation Techniques

X-ray diffraction has been the method of choice to determine the structure of crystalized proteins with resolution well below 2 Å. In systems where AMPs co-crystalize with their target, X-ray can provide high-resolution structures, as demonstrated by the recent structures of peptides complexed with *Pseudomonas aeruginosa* protein LecB (5). The requirement of a crystalline environment, however, has excluded this technique from in-cell studies, which has limited our understanding of the impact of the cellular environments on intricate interactions. This hurdle is gradually being phased out with the development of soft X-ray tomography, a fast progressing field, for generating high-resolution images of cellular systems in a similar fashion to cryo- electron microscopy (EM) tomography. Indeed, recent images of cells at 30–50 nm resolution have been reported (6, 7).

Cryo-EM has drastically improved the capability to determine the structure of proteins with <3 Å resolution, as recently reported by Herzik et al. (8). This imaging technique is particular well-suited for cellular environments and large complexes since labeling is not required for signal detection and is not impeded by conformational heterogeneity (9, 10). For instance, the structures of bacterial ribosomes have been investigated as they are good candidates for drug targeting (11). Cryo-EM can also provide a wealth of information on the topology of cell structures, such as the architecture of organelles at membrane contact sites (12) or how membrane-active molecules, such as AMPs, can alter the cell morphology (13). However, high-resolution cryo-EM of peptides and proteins is often limited if X-ray or NMR derived structures are not available to assist computer-guided structural fitting of images, known as single particle analysis. Furthermore, cryo-EM is unable to provide information on the dynamic interactions between biomolecules such as receptor-ligand interplays. Ultimately, in-cell structural studies of membrane-active peptides and small membrane proteins remain rare and difficult, mainly due to their size.

Imaging using fluorescent probes has also been successful in providing structural details of cellular components. Resolution is constantly improving with the latest single particle methodology at the forefront (14). In-cell fluorescence has a severe limitation, however, due to the necessity of a fluorescent probe which is invasive and challenging to target in a crowded cellular environment.

NMR, and in particular ssNMR, is well suited to tackle in-cell studies as, like EM, it is not limited by the nature of the environment and does not require a perturbing label for signal detection. Notably, the majority of the AMP structures deposited in the RSCB Protein Data Bank (PDB) have been determined by solution-state NMR, albeit in membrane mimetics or mixed solvents. In-cell NMR has provided the first structure of a protein at atomic resolution in an intact cellular environment (15). In addition to its capability to provide high-resolution structures, NMR is particularly powerful for investigating dynamics, which opens a window to view the mechanism of complex *in situ* physiological processes. NMR has provided deep insights into protein-protein, protein-lipid, and protein-ligand interactions, with particular success in monitoring transient interactions (16). NMR is the most powerful high-resolution technique for determining binding constants, folding thermodynamics and kinetics of biomolecular interactions. However, NMR has rather low sensitivity and background signals can contribute significantly: difficulties that impose long experimental times compounded by limited cell viability. These practical challenges and ways around them for in-cell NMR studies will be discussed in the following section.

## In-Cell Solid-State NMR

### Practical Challenges

A key physical property necessary for high-resolution NMR is the rapid reorientation, or rotational tumbling, of the macromolecules within the magnetic field. Large molecules tumble slowly and display severely broadened NMR signals, which could become undetectable in solution-state experiments. Indeed, most cellular components are not detected by solution-state NMR although, interestingly, the viscosity of the intracellular environment is only moderately higher (1.2–2-fold) than the usual buffers used *in vitro* (17, 18). In fact, it has been determined that weak and non-specific interactions that slow down molecular motions of soluble molecules—termed quinary structures (19)—are mostly responsible for the absence of these NMR signals (20).

ssNMR is not limited by slow molecular motion, which places this non-invasive technique as ideal for investigating the interplay between AMPs and bacteria, fungi or other cell systems. However, the intrinsic slow molecular tumbling combined with the strong anisotropy lead to lower sensitivity in comparison to solution-state NMR. Thus, greater amounts of material and longer experimental times would be necessary to obtain structural information with high resolution. ssNMR uses magic angle spinning (rotation of the sample at 54.74° relative to the magnetic field) to partially reintroduce motional averaging thereby providing enhanced resolution. However, to fully average the broadening interactions with the magnetic field, such as the dipolar couplings or the chemical shift anisotropy, the rotor must be spun above 110 kHz for solid samples. This is achievable with latest NMR probe developments with rotors of 0.9 mm diameter but remains limited to particular samples, such as microcrystals. This highlights a limiting factor in ssNMR as the sample volumes are generally well below 100 μL, which is a challenge for live cell experiments. Indeed, most of the sample volume will be occupied by the aqueous phase, resulting in less signal from molecules of interest, e.g., lipids. Furthermore, cellular background is usually significant in ssNMR since all molecules, regardless of their tumbling rate, are contributing to the NMR signal. However, filtering techniques using specific pulse sequences can be used to select rigid (Hartmann-Hahn cross-polarization transfer, CP) vs. mobile (insensitive nuclei enhanced by polarization transfer, INEPT) molecules (21), opening strategies to design NMR experiments.

In addition to the spectral broadening due to the biomolecular tumbling rates, other factors contribute to reducing the resolution of the NMR signals of cellular components. The heterogenous environments in cells induce gradients of magnetic susceptibility and cellular degradation is changing the signal during acquisition. It becomes apparent that fast signal acquisition and/or signal enhancement are important challenges to be tackled in order to develop in-cell ssNMR further. The development of dynamic nuclear polarization (DNP) has provided tremendous NMR signal enhancement, requiring spin labels and cryogenic conditions, thereby enabling in-cell studies of peptides and proteins while cell integrity is preserved under cryoprotection during experimental time (22). Many challenges remain before in-cell studies using DNP-enhanced NMR techniques become routine, mainly due to lower signal resolution at cryogenic temperatures (100 K). Still, the technology is rapidly improving with reduced rotor sizes for faster spinning speed and development of DNP at high magnetic fields (23). A recent review on DNP NMR of large biomolecular assemblies has emphasized that cellular preparations do not provide as high signal enhancements asmi crocrystals or precipitates and require particular isotopic enrichment to alleviate the background signal (24).

**Figure 1:**
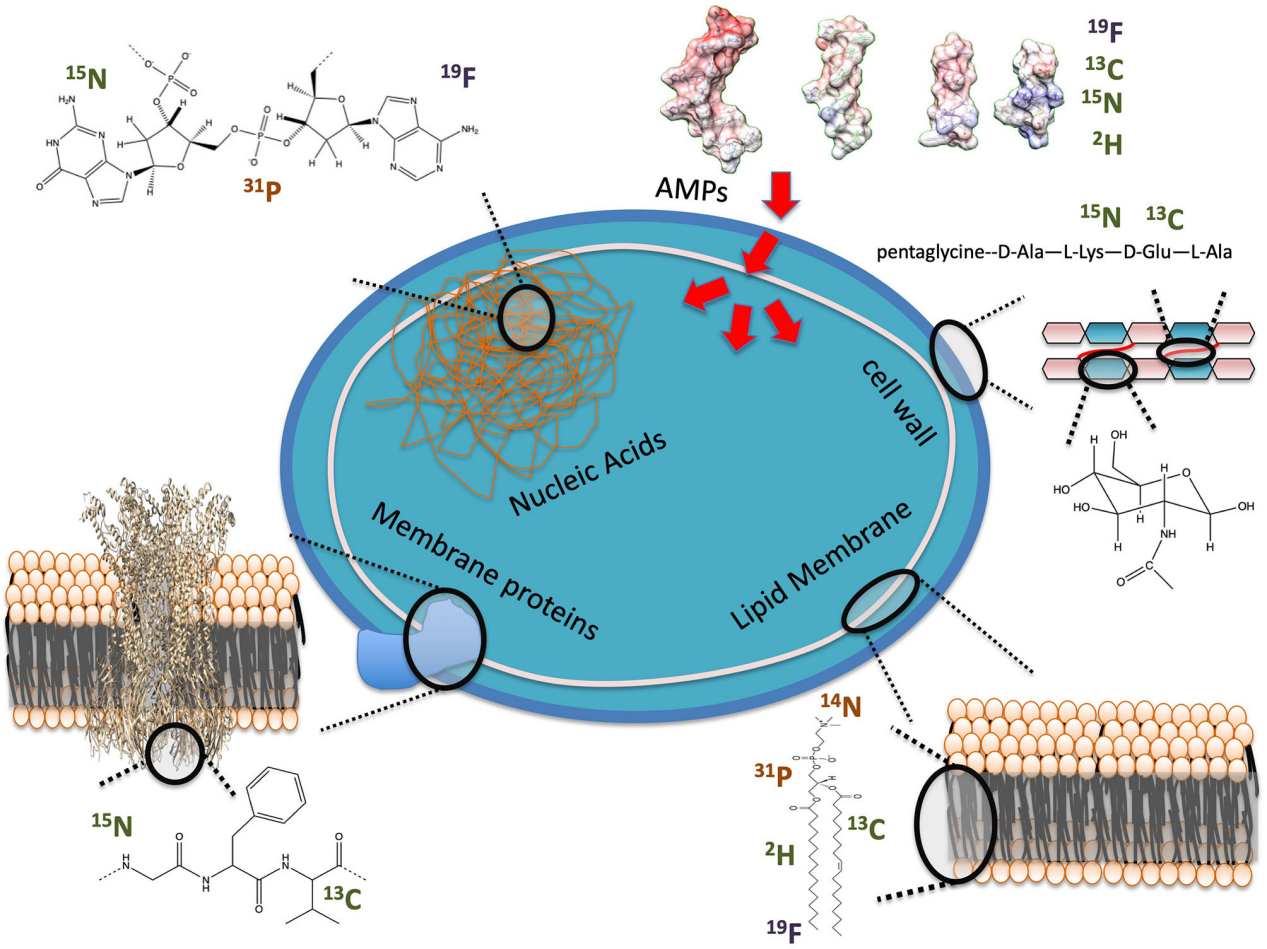
Examples of NMR observables for in-cell studies of AMP interactions with bacteria. Nuclei are labeled as present in cells at high (orange) or low (green) natural abundance or unnatural (blue).

NMR studies of naturally abundant metabolites and molecules with high copy levels have provided useful *in vivo* details of physiological processes, albeit with a limited resolution and greater complexity of signal interpretation, which often requires heavy statistical analysis, such as principal component analysis (25). In-cell NMR differs as it focuses typically on a specific molecule with high resolution. To achieve this specificity, the molecule of interest needs to be labeled with isotopes that are otherwise in very low abundance at natural level or not present in biomolecules. ^13^C (1.1% natural abundance), ^15^N (0.4% natural abundance), ^2^H (0.01% natural abundance) and ^19^F (100% natural abundance but not found in biological environments) have been successfully incorporated into biomolecules to provide specific monitoring within complex environments (Figure 1). Procedures are now commonly available to design labeling schemes for proteins over-expressed in prokaryotic cells (26) and less so in eukaryotic cells, although methodology to deliver labeled ubiquitin into mammalian cells recently was achieved for in-cell NMR studies (27). The production of isotopically enriched recombinant AMPs for structural studies has not been extensively reported, but a similar strategy could be applied and has been for commercial production of several AMPs (28). Synthetic methods have also been used to introduce selective labeling within peptides, but this remains a costly method that is often limited to labeled hydrophobic amino acids. A few studies have reported the expression of AMPs in decent yields, by using a fusion partner thereby limiting the toxicity toward the host system and post-translation modification or degradation (29, 30). It is noteworthy that AMPs are often naturally C-amidated, which confers higher stability from serum degradation. One expression system attempted to generate C-amidated AMPs by using an intein fusion tag. The self-splicing tag is removed under reducing conditions and high concentration of ammonium bicarbonate, thereby introducing the amide group at the cleavage site (31). An issue of the method, however, is the low final yield as the fusion tag can easily represent 90% of the expressed fusion protein molecular weight, which drastically reduces the amount of recovered peptide, and additional purification of the carboxylic vs. amidated C-terminus peptides may be required.

As seen in Figure 1, several potential targets by AMPs can be monitored by ssNMR using either naturally abundant reporters, such as ^31^P, or by enriching cellular components with isotopes, such as ^2^H. Phosphorus is naturally found in high content as phosphate moieties are used as a building block in phospholipids but also in nucleic acids (NA) and other metabolites such as ATP. ^2^H has been introduced into bacterial phospholipids by feeding bacteria with ^2^H labeled fatty acids (32, 33). Cell wall components have also been labeled by introducing ^13^C, ^15^N and even ^19^F into the peptidoglycan structure (34). Peptides and proteins can be ^13^C, ^15^N and even ^2^H isotopically enriched to improve sensitivity and resolution of NMR studies (26, 30).

Overall, ssNMR studies of AMPs in whole bacterial cells have been scarce due to the necessity of introducing biochemical labeling steps. However, recent studies have demonstrated that in-cell NMR can provide new insights into the mode of action of AMPs, not otherwise accessible within *in vitro* environments.

### AMPs' First Encounter, the Gram-Positive Cell Wall or the Gram-Negative LPS Layer

Bacteria have a protective external layer around their phospholipid membranes: the cell wall for Gram-positive and the lipopolysaccharide layer (LPS) for Gram-negative bacteria. Knowledge of these structures is important for understanding how AMPs either get through to target the phospholipid membranes or how they inhibit cellular processes by remaining in these external layers.

**Figure 2:**
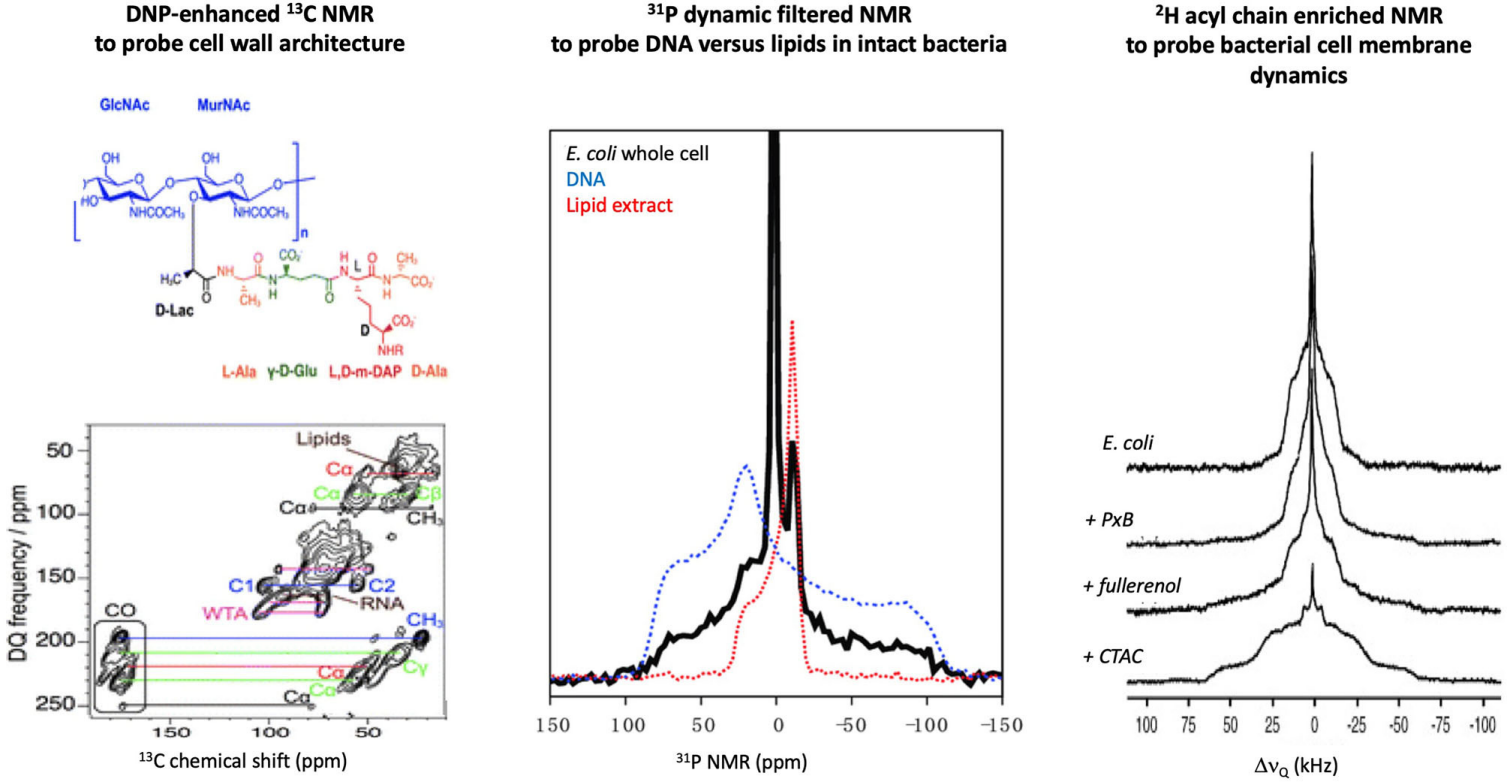
Examples of in-cell solid-state NMR studies of intact bacteria. **(Lef)t** Using DNP-enhanced ^13^C NMR, the cell wall of *B. Subtilis* was investigated and signals from several cell components could be assigned, which allowed the impact of AMPs on these structures to be monitored [adapted from (35)]. **(Middle)** ^31^P NMR of *E. coli* can differentiate DNA vs. lipid signals due to the difference in intramolecular dynamics, which allowed direct monitoring of AMP impact on cell membranes or secondary targets such as DNA [adapted from (36)]. **(Right)** ^2^H NMR of *E. coli* fed with ^2^H enriched fatty acids has allowed monitoring the effect of membrane active molecules (PxB, polymyxin B; fullerenol, nanoparticle; CTAC, cetyltrimethylammonium chloride) on the dynamic of the bacterial membranes [adapted from (32)].

The cell wall is made of a peptidoglycan (PGN) sacculus anchored within phospholipid membranes by teichoic acids. ^13^C ssNMR studies of *Staphylococcus aureus* and *Bacillus subtilis* (Figure 2) have determined that the PGN mesh structure is formed by the disaccharide backbone four-fold screw helical symmetry with each PGN stem oriented 90° from the previous stem (37). Most AMPs are not retained by the PGN mesh partially due to the neutral charge of the structure vs. the highly negatively charged surface of the Gram-positive phospholipid membranes. Yet, AMPs, such as the human cationic polypeptide ECP (eosinophilic cationic protein), have shown strong interactions with the PGN, which interfere with the cell replication process (38). Cegelski *et al*. have reported methods to label the PGN of Gram-positive bacteria and have monitored the binding of antibiotics to the cell wall of *S. aureus* (34). A similar achievement was obtained using the DNP ssNMR approach where lipid II binding antibiotics were investigated *in situ*. The importance of a native environment was highlighted for pore formation of nisin with increased plasticity of the peptide observed in native bacteria (39).

The outer leaflet of the outer membrane (OM) of Gram-negative bacteria is mainly made of LPS, whose complex structure is species specific (40). LPS can contain various amount of phosphate and pyrophosphate groups, which can modulate the negative charge density, and thus electrostatic interactions with cationic AMPs (41). NMR has provided a wealth of structural details on LPS incorporated into micelles (42) and isolated OMs have been used to investigate AMP interactions with LPS (43). These studies have demonstrated that external LPS often limits the activity of AMPs by strongly binding to the peptides thereby preventing interior access to Gram-negative bacteria. Yet, high-resolution in-cell NMR studies of intact Gram-negative bacteria remain to be performed so as to specifically address AMP interactions with the complex LPS layer under native conditions. This may require specific labeling of the LPS to enable signal separation between it and other intracellular molecules such as the phospholipids.

### Bacterial Lipid Membrane, the Main Target for AMPs

AMPs have displayed a remarkable affinity for negatively charged membranes of bacteria vs. neutral eukaryotic membranes (44). ^31^P is a natural highly abundant nucleus that can report on the architecture of the lipid bilayers and the dynamics of the lipid headgroup (45). A recent ^31^P NMR study of intact *Escherichia coli* (36) has reported that the AMP maculatin 1.1 increases the dynamics of the membrane lipids, which is direct evidence of the disruptive effect that AMPs trigger in membranes of live bacteria.

Not only the charge but also the nature of the lipid acyl chains has been shown to modulate AMPs activity. Incorporation of ^2^H labeled fatty acids into the growth media of bacteria has allowed specific monitoring of AMPs perturbation of the hydrophobic core of bacterial (32, 33). ^2^H NMR spectra can provide a more direct measure of how deep AMPs perturb the dynamics along the lipid acyl chain as methyl deuterons found at the chain terminus and deuterons near the glycerol region at the water interface are usually well resolved (Figure 2). Although a small subset of AMPs and bacteria have been investigated by in-cell ^2^H NMR, similar observations have been reported, i.e., AMPs increase the acyl chain dynamics by inserting into the hydrophobic core of the bacterial membranes. However, the degree of perturbation of *E. coli* vs. *B. subtilis* for a series of AMPs having different charges and lengths was quite disparate, which supports the importance of studying the impact of AMPs *in situ* (46, 47).

### Intracellular Targets, AMPs Secondary Targets?

^31^P NMR studies of *E. coli* bacteria with the AMP maculatin 1.1 revealed that the phospholipid membranes were significantly perturbed but, unexpectedly, that DNA packing was also impacted (36). NMR is able to filter between the rigid DNA and the mobile phospholipid membrane which allowed the multiple effects induced on the bacteria under the AMP action to be monitored (Figure 2). This study showcased that AMPs can have multiple targets and, unlike *in vitro* systems, by monitoring the entire cellular response, the full spectrum of the bactericidal mechanism may be tracked.

### AMPs Structure and Self-Assembly in Bacteria: the Key to Understanding AMPs Mode of Action

Most cationic linear AMPs are unstructured in an aqueous environment but adopt a secondary structure when in contact with a lipid membrane (48). Do these *in vitro* observations hold in bacteria? To obtain the *in situ* structure of an AMP, higher amounts of ^13^C and ^15^N labeled peptides and longer experimental times are necessary to extract the intricate dipolar network of the amino acid residues. This is a hurdle since labeling is tedious (and costly) and long experimental times are detrimental for cell integrity. DNP-NMR has opened new possibilities to tackle these practical issues. By significantly enhancing the NMR signal using spin labels under cryogenic conditions, in-cell DNP-NMR experiments of *E. coli* incubated with ^13^C,^15^N synthetically enriched labeled AMPs at specific residues is achievable. The ^13^C to ^15^N atomic distances obtained by REDOR NMR (49, 50) are used to provide restraints for structure calculation, i.e., determining if AMPs retained the expected helical pitch between residue i to residue i + 3 in bacteria (51). Once large-scale expression systems for C-amidated AMPs are optimized, AMP structures and peptide-peptide contact maps will be achievable by in-cell solid-state DNP-NMR studies.

## Perspectives

Understanding the mode of action of AMPs based on their primary sequence, deciphering their self-assembly mechanism and tracking their interactions with multiple intracellular targets in intact bacteria are crucial in order to develop new therapeutics. Although at an early stage, in-cell ssNMR has demonstrated the capability to provide important structural details, such as how cell membranes and/or DNA of bacteria respond to AMPs. This additional knowledge will give rise to further questions and in turn stimulate new biochemical engineering that further will extend *in situ* ssNMR studies. From the production of isotopically enriched AMPs to identifying specific NMR signals in bacteria, ssNMR offers exciting prospects to image the interplay of AMPs with bacteria and plays an important role in complementing other imaging techniques and biochemical assays.

## Author Contributions

M-AS, FS, and DK conceived and wrote the paper. All authors contributed to the article and approved the submitted version.

## Conflict of Interest

The authors declare that the research was conducted in the absence of any commercial or financial relationships that could be construed as a potential conflict of interest.
